# Robotic approach to vesicourethral anastomotic stenosis and resection of remaining prostate after radical prostatectomy

**DOI:** 10.1590/S1677-5538.IBJU.2022.0249

**Published:** 2022-06-10

**Authors:** Diego Moreira Capibaribe, Natália Dalsenter Avilez, Carlos Alberto Ricetto Sacomani, Alexandre Sá Pinto da Nobrega Lucena, Leonardo Oliveira Reis

**Affiliations:** 1 Universidade Estadual de Campinas - UNICAMP Faculdade de Ciências Médicas da UroScience Campinas SP Brasil UroScience, Faculdade de Ciências Médicas da Universidade Estadual de Campinas - UNICAMP - Campinas, SP, Brasil; 2 AC Camargo Cancer Center Sao Paulo SP Brasil AC Camargo Cancer Center - Sao Paulo, SP, Brasil; 3 Universidade de Fortaleza UniFor Fortaleza CE Brasil Universidade de Fortaleza, UniFor, Fortaleza, CE, Brasil; 4 Pontifícia Universidade Católica de Campinas - PUC Campinas SP Brasil Pontifícia Universidade Católica de Campinas - PUC -Campinas, - Campinas, SP, Brasil

## Abstract

**Objective:**

To show a total transabdominal robotic approach to an extensive recalcitrant vesicourethral anastomotic stenosis (VUAS) after open radical prostatectomy (ORP) with end-to-end anastomosis. While there is very little literature on the matter and even fewer videos showing the actual surgical view with a step-by-step explanation in complex cases, VUAS robotic transabdominal surgery provides better view and reach, with potentially better continence results, without the need for pubectomy.

**Methods:**

A 72-year-old male was submitted to a failed ORP for Gleason 3+4 localized cancer 2 years before, where the wrong plane of dissection left behind prostate remnants and the seminal vesicles, which evolved with a complex stenosis and recurrent episodes of acute urinary retention (AUR) that started two weeks after the first catheter removal. Five endoscopic procedures in total were unsuccessful and AUR reoccurred. A vesico-urethral cystography (VUC) and multiparametric prostate and urethral MRI found the seminal vesicles with prostate remnants, two centimeters urethral stenosis from bladder neck to bulbar urethra and periurethral fibrosis with no evidence of residual tumor. PSA was 1.2 and prostate biopsy showed no tumor on prostate remnant. A transabdominal robotic approach was chosen.

**Results:**

Prostate residue, bladder neck and periurethral fibrosis were excised, with healthy mucosa found on both ends. End-to-end anastomosis was successful. Drain and catheter were removed on the 1st and 14th post-operative day, respectively, with good urinary stream. A VUC at 30 days showed a patent bladder neck. Incontinence was 3 pads/day after catheter removal and decreased to 1 pad/day after 180 days.

**Conclusions:**

VUAS may reach 15% ( 1 , 2 ) and endourologic therapies are first-line choices, however, recalcitrant cases require reconstruction ( 3 – 6 ). The most common approach is perineal, with high incontinence rates, reaching >90% ( 7 , 8 ). The retropubic alternative has better but also discouraging numbers of up to 58% incontinence rates ( 9 ). Though with 100% social continence results, the 2021 European guidelines still could not recommend the robotic procedure as standard of care due to evidence limited to anecdotal reports ( 10 – 12 ).

**Available at:** http://www.intbrazjurol.com.br/video-section/20220249_Reis_et_al

## References

[1] Wilt TJ, Jones KM, Barry MJ, Andriole GL, Culkin D, Wheeler T (2017). Follow-up of Prostatectomy versus Observation for Early Prostate Cancer. N Engl J Med.

[2] Modig KK, Godtman RA, Bjartell A, Carlsson S, Haglind E, Hugosson J (2021). Vesicourethral Anastomotic Stenosis After Open or Robot-assisted Laparoscopic Retropubic Prostatectomy-Results from the Laparoscopic Prostatectomy Robot Open Trial. Eur Urol Focus.

[3] Wessells H, Angermeier KW, Elliott S, Gonzalez CM, Kodama R, Peterson AC (2017). Male Urethral Stricture: American Urological Association Guideline. J Urol.

[4] Giannarini G, Manassero F, Mogorovich A, Valent F, De Maria M, Pistolesi D (2008). Cold-knife incision of anastomotic strictures after radical retropubic prostatectomy with bladder neck preservation: efficacy and impact on urinary continence status. Eur Urol.

[5] Eltahawy E, Gur U, Virasoro R, Schlossberg SM, Jordan GH (2008). Management of recurrent anastomotic stenosis following radical prostatectomy using holmium laser and steroid injection. BJU Int.

[6] Lagerveld BW, Laguna MP, Debruyne FM, De La Rosette JJ (2005). Holmium:YAG laser for treatment of strictures of vesicourethral anastomosis after radical prostatectomy. J Endourol.

[7] Simhan J, Ramirez D, Hudak SJ, Morey AF (2014). Bladder neck contracture. Transl Androl Urol.

[8] Reiss CP, Pfalzgraf D, Kluth LA, Soave A, Fisch M, Dahlem R (2014). Transperineal reanastomosis for the treatment for highly recurrent anastomotic strictures as a last option before urinary diversion. World J Urol.

[9] Nikolavsky D, Blakely SA, Hadley DA, Knoll P, Windsperger AP, Terlecki RP (2014). Open reconstruction of recurrent vesicourethral anastomotic stricture after radical prostatectomy. Int Urol Nephrol.

[10] Dinerman BF, Hauser NJ, Hu JC, Purohit RS (2017). Robotic-Assisted Abdomino-perineal Vesicourethral Anastomotic Reconstruction for 4.5 Centimeter Post-prostatectomy Stricture. Urol Case Rep.

[11] Kirshenbaum EJ, Zhao LC, Myers JB, Elliott SP, Vanni AJ, Baradaran N (2018). Patency and Incontinence Rates After Robotic Bladder Neck Reconstruction for Vesicourethral Anastomotic Stenosis and Recalcitrant Bladder Neck Contractures: The Trauma and Urologic Reconstructive Network of Surgeons Experience. Urology.

[12] Lumen N, Campos-Juanatey F, Greenwell T, Martins FE, Osman NI, Riechardt S (2021). European Association of Urology Guidelines on Urethral Stricture Disease (Part 1): Management of Male Urethral Stricture Disease. Eur Urol.

